# Primary mucinous cystadenocarcinoma of renal pelvis: a case report

**DOI:** 10.1186/1757-1626-2-9395

**Published:** 2009-12-23

**Authors:** Mehdi Fareghi, Afshin Mohammadi, Kazem Madaen

**Affiliations:** 1 Urology Department, Imam Reza University Hospital, Tabriz University of Medical Sciences, Tabriz, Iran; 2 Radiology Department, Imam Khomeini University Hospital, Urmia University of Medical Sciences, Urmia, Iran

## Abstract

**Background:**

We report a case of primary mucinous cystadenocarcinoma of renal pelvis which radiologically resembled large multicystic mass in 45 years old man.

**Case Presentation:**

The patient referred to our center with loin pain and progressive abdominal distention from 4 years ago. In the previous published literature, four cases of mucinous cystadenocarcinoma of renal origin have been published. Abdominal CT showed complete replacement of left kidney by a large multiloculated cystic mass accompanied with multiple large nephrolithiasis. Nephrectomy was performed and histopathology revealed covering of epithelium of renal pelvis by columnar epithelium and scattered goblet cells and mucous gland.

**Conclusion:**

Mucinous cystic neoplasms of kidney are rare entity and our case and few similar reported cases showed that this tumor is an unique clinicopathologic renal mass that may be classified by World Health Organization classification in the future

## Background

The most common neoplasms in the renal pelvis are urothelial in origin, although infrequently, squamous and glandular neoplasm may arise within the renal pelvicalyceal system through metaplastic transformation of the urothelium due to long-standing obstruction and infection and chronic irritation by urolithiasis [1]. In previous published literature invasive mucinous adenocarcinoma has been well documented [2,3], but malignant mucinous cystic tumors are exceedingly rare primary neoplasms involving renal pelvicalyceal system with only 4 cases reported in the literature [4-7].

Because knowledge about renal mucinous cystic neoplasms is extremely limited and they have not been recognized in the World Health Organization classification of pelvicalyceal system malignancy. We describe the clinical, radiological and pathological findings of a 45 years old man with mucinous cystadenocarcinoma arising in a background of extensive mucinous metaplasia of pelvicalyceal urothelium secondary to probably chronic nephrolithiasis and hydronephrosis.

## Case Presentation

A 45-year-old man presented with a history of a dull aching pain in the left flank and progressive abdominal distention since 4 years ago. Recently the patients complain from aggravation of abdominal pain and new onset constipation. He had no history of passing stones. He had not history of haematuria, dysuria, frequency or fever during 4 years ago.

**Figure 1:**
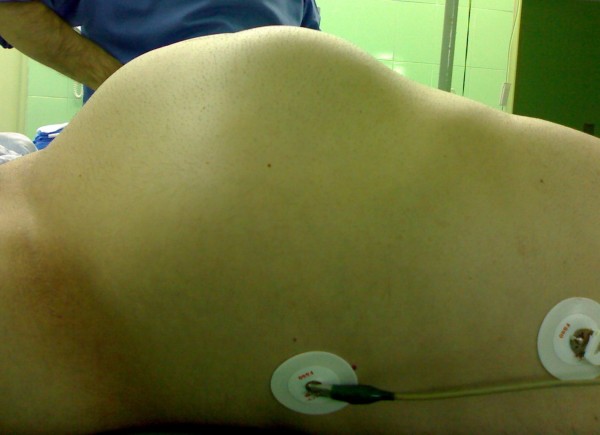
**Image show severe abdominal distention of a 45 year's old man**.

General physical examination revealed nothing positive. Abdominal examination revealed asymmetrical buldging of abdomen on inspection and a firm fixed mass in the left hypochondrium that extended to the epigastrium and hypochonderium on palpation. (Figure 1)

Laboratory investigations showed haematuria, mucosuria and 10-12 pus cells/high power filed in urine examination. Urine culture was negative.

Routine hematological investigations were within normal limit.

Intravenous pyelography revealed the non-functioning left kidney with multiple large opaque stones. In transabdominal sonography a huge cystic mass with internal echo debris and multiple stones without remarkable renal parenchyma was observed that mimicked sever long-standing hydronephrosis accompanied with pyonephrosis.

**Figure 2:**
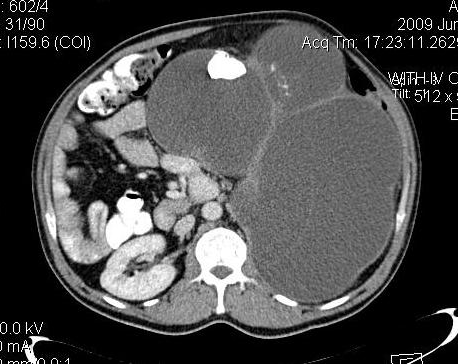
**Axial image show huge multiloculated cystic mass with a large nephrolithiasis and thick septation**.

Abdominal CT scan (64-MDCT, Siemens. Somatom, Sensation) with IV and Oral contrast revealed a huge lobulated multicystic thin wall mass measuring 25*20*30 cms with thick septation accompanied with solid component without marked enhancement and some large nodular calcified density (stone) that completely replaced left kidney. (figure 2).

Abdominal CT did not reveal any other mass lesions or ascites in the abdomen. There was no singe of hydroureter in abdominal sonography and abdominal CT scan.

**Figure 3:**
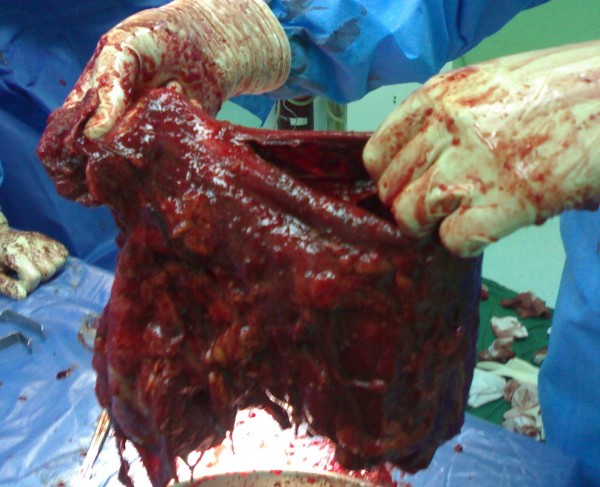
**Photograph show gross appearance of a complex multiloculated cystic mass replacing the entire left kidney**.

Surgical excision of the mass was performed completely. Grossly left kidney was completely replaced by lobulated multicystic mass measuring 30*20*20 cms that were filled with necrotic and mucinous material. (Figure 3).

**Figure 4:**
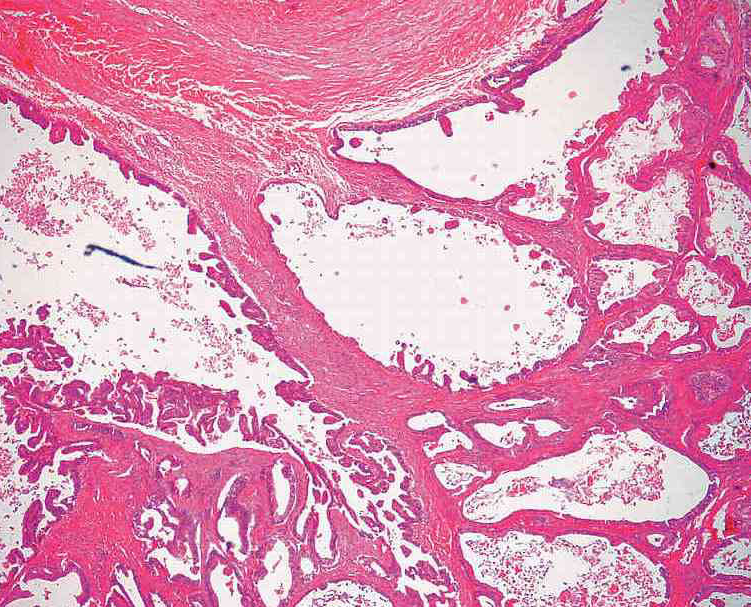
**Cystadenocarcinoma showing variously sized cystic structures lined by atypical, but well-differentiated mucin-producing glandular epithelium, focally forming papillary structures**.

Histopathology revealed that the tumor was composed of single layer of columnar epithelium with scattered goblet cells and mucous glands resemblance to intestinal mucosa with abundant extracellular mucin compatible with cystic mucinous adenocarcinoma (Figure 4).

The renal capsule, Ureter and renal hilar vessels were intact (T 3).

## Discussion

Tumors of the renal pelvis are uncommon, with relative frequency of transitional cell carcinoma (90%), squamous cell carcinoma (10%) and adenocarcinoma (1%) [1]. Mucinous cystic neoplasms of kidney are exceedingly rare primary neoplasm, with only 5 benign, 3 borderline [7] and 4 malignant [4-7] cases reported in the literature. Mucinous cystadenocarcinima of renal pelvis was first described in 1960 by Hasebe et al.

These tumors occur as a result of metaplasia of the transitional epithelium of the calyces and pelvis into glandular epithelium, which then undergoes a malignant transformation [8]. Presence of chronic infection, hydronephrosis and calculi are commonly associated with these tumors [9]. Our patient had multiple large stone predisposing him to all the above conditions. Some authors postulated that formation of the calculi might be the result of over secretion of glycoproteins by the tumor and binding of that with cations such as sodium, calcium, and magnesium, forming larger calculi. Thus, calculi may not be the cause of the neoplasm [10].

Patients are often asymptomatic. Hematuria is the most common presenting sign and loin pain and palpable abdominal mass are the late presentation of this tumor[7]. Our patient had no history of gross or microscopic hematuria.

Radiological studies may not be able to identify malignant tumor [1]. In our patients neither ultrasonography nor abdominal CT scan were not suggestive for malignancy. According to imaging finding long-standing UPJO and chronic hydronephrosis due to nephrolithiasis was the most probable diagnosis of our patient. Because mucinous cyst adenocarcinoma is an exceedingly rare entity a careful search for a primary carcinoma originating elsewhere such as pancreas, ovary and appendix should be excluded. In our patient the patient was male and radiologic evaluation of pancrease and appendix was negative for malignancy.

Mucinous cystic neoplasms of kidney are rare entity and our case and few similar reported cases shwe that this tumor is an unique clinicipathologic renal mass that may be classified by World Health Organization classification in the future.

## Consent

Written informed consent was obtained from the parents of the patient for publication of this case report and accompanying images. A copy of the written consent is available for review by the Editor-in-Chief of this journal.

## Competing interests

The authors declare that they have no competing interests.

## Authors' contributions

MF and KM were the attending doctor, carried out the surgical procedure and literature review. AM was the attending doctor and collected the data and literature review, and wrote the manuscript. All authors read and approved the final manuscript.
